# Statistical Models in Quality Control for Trace Analysis

**DOI:** 10.6028/jres.093.023

**Published:** 1988-06-01

**Authors:** Robert S. Elder

**Affiliations:** FSIS Science, Room 612 Annex, U.S. Department of Agriculture, Washington, DC 20250

## 1. Introduction

It is well known that there are important factors affecting accuracy in trace analysis, such as handling loss, contamination and purity of reagents. The contention of this paper is that the statistical model for analytical error is another important factor, that currently is receiving much attention. A normal distribution is the model upon which the statistical procedures used in laboratory quality control (QC) customarily are based. However, examinations of certain analytical procedures and results of trace analyses reveal features that are inconsistent with normality.

## 2. Alternate Models

The three models to be discussed are the normal distribution, the lognormal distribution, and no distribution. There are good reasons for expecting one of these models to be appropriate in many circumstances.

The normal distribution is symmetric, bell-shaped, and of unbounded range. It is characterized by two independent parameters, its mean (*μ*) and standard deviation (*σ*), which can be directly associated with analytical bias and precision. One reason for the widespread applicability of this model is the Central Limit Theorem, the practical result of which is that sums of random variables tend to be normally distributed under mild conditions. Based on the Central Limit Theorem, it has been said that “in the case of a well devised analytical system of measurement and a properly performed analysis… analytical results will be normally distributed or, at least, almost so” [1]. This statement assumes that analytical errors are additive.

The lognormal distribution [2] is asymmetric, bounded below by zero, and is defined by the function

$$f\left(x\right)=\exp\left[-{\left(\log\left(x\right)-\alpha \right)}^{2}/2\delta^{2}\right]/{\left(2\pi \delta^{2}x\right)}^{1/2}$$

The distribution takes its name from the fact that if *x* is lognormally distributed, log(*x*) is normally distributed. The mean and standard deviation of the distribution are *μ*=exp(*α+*1/2*δ*^2^) and *σ*=*μ·*(exp(*δ*^2^)−1)^1/2^; thus the coefficient of variation (*CV*) is constant with respect to the mean. For QC use one can reparameterize the model to make the mean and coefficient of variation the basic parameters (by defining *δ*^2^=log[1+(*CV*/100)^2^] and *α*=log(*μ*)−*δ*^2^/2). In addition, the model can be generalized to shift its origin from zero to *γ* by taking log (*x*+*γ*) to be normally distributed.

**Figure 1:**
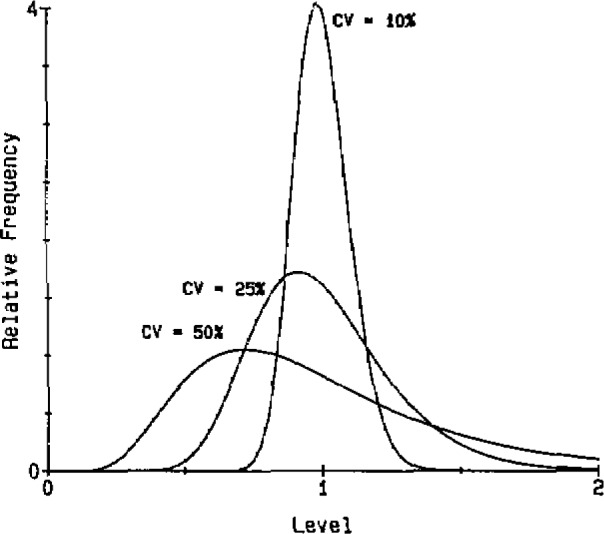
Lognormal distributions.

Figure 1 illustrates how the skewness of the distribution increases as the *CV* increases. For *CV*s of less than 10%, the model differs very little in shape from the normal distribution. (This is another reason for the usefulness of the normal model.)

**Figure 2:**
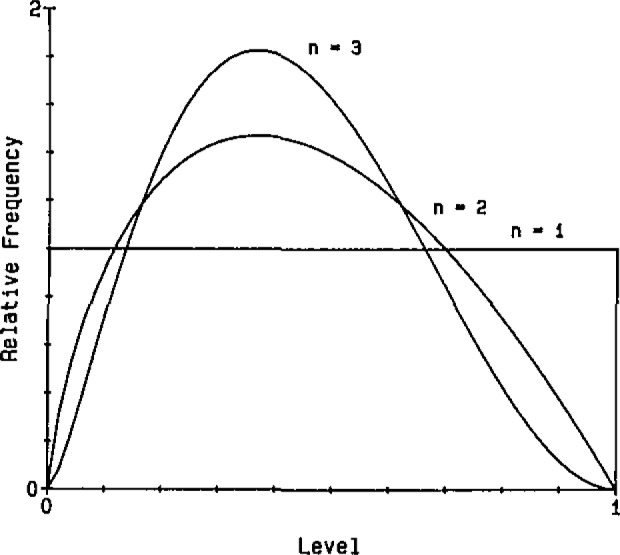
Distributions of geometric means of samples from a rectangular distribution.

Based on the Central Limit Theorem and the fact that the logarithm of a product of random variables equals the sum of the logarithms of the random variables, the lognormal model tends to be appropriate for multiplicative processes. Figure 2 shows how rapidly the distribution of a product of random variables can approach the lognormal distribution as the number of factors (n) increases.

The third alternative, no distribution, is the most likely model in the absence of effective quality control. The first job before applying statistical models and procedures is to get a distribution. “Stability, or the existence of a system, is seldom a natural state. It is an achievement…” [3]. Producing a consistent distribution requires serious effort from design (method development) through production (routine analysis).

## 3. Choosing the Right Model

One way to decide what statistical model to use is to turn to the technical literature for guidance. However, many sources do not address the subject explicitly (e.g., see the ACS Principles of Environmental Analysis [4]), and others give conflicting advice. For example, the Statistical Manual of the AOAC [5] says: “It is understood that random errors are equally likely to be positive or negative and to vary in size in a manner that is adequately described by the normal law of errors.” Eckschlager and Stepanek [6] say that a shifted lognormal distribution is appropriate for concentrations above the determination limit. Thompson and Howarth [7] claim to make the “case against” the lognormal distribution.

A second approach to choosing a model is to look at data. Unfortunately, there is seldom enough data to reach a conclusion [7], or the data is messy (contains blunders and outliers and is censored below the limit of quantitation). Nevertheless, one can find clues in QC data; for example, appropriateness of *CV* and percent recovery as summary statistics hint at a multiplicative process and log-normality. The work of Horwitz and colleagues in relating analytical precision to concentration across many analytical methods is interesting in this regard: it shows the widespread usefulness of the *CV* as a measure of imprecision, and shows how the total *CV* increases as the level of applicability of methods decreases [8]. At the ppb level, the Horwitz model gives a *CV* of about 45%.

The third and best method of choosing a model is to combine whatever knowledge can be obtained from data with what can be deduced from the nature of the measurement process. Is the process additive or multiplicative? There are certain common steps in sample preparation, such as concentration, dilution and extraction, that are multiplicative in nature [9,10]. An example of an analytical method with a multiplicative quantitation process is the GC/MS method for wastewater analysis [11]: the test result is a product or quotient of response factor, concentration of internal standard, peak areas, and volume of original sample. Coupled with the fact that *CV*s for this method are as high as 50%, there is good reason to expect the lognormal model to be appropriate.

## 4. Impact of the Lognormal Model

**Figure 3:**
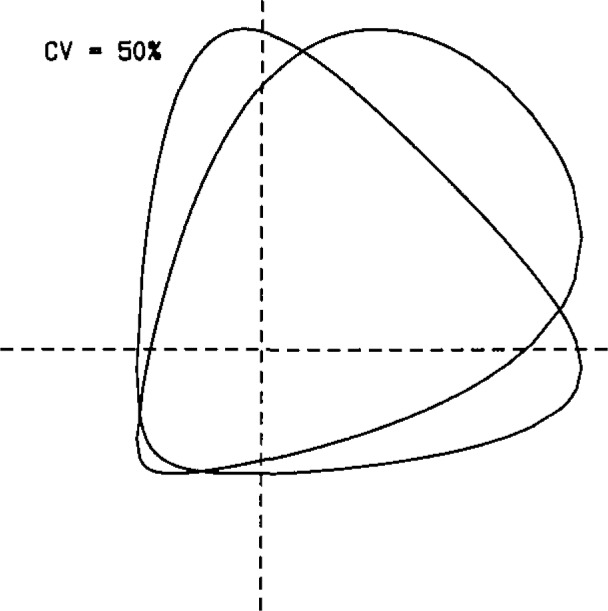
Expected configurations of Youden two-sample charts for lognormal data.

A legitimate concern with the lognormal model is how it will affect traditional concepts and procedures of analytical QC. The answer is that the impact ranges from negligible to serious depending on the procedure involved. Consider these examples:

1. Repeatability interval. The distribution of the difference of two identically distributed lognormal random variables is well approximated by the normal distribution. Therefore, the impact of lognormality on this concept is negligible.
2. Control charts. It is not necessary to work with log-transformed data to control either bias or precision. For example, bias can be monitored with a percent recovery control chart [12], and within-laboratory precision by a chart for the ratio of duplicate measurements. It becomes more important to base control limits on the lognormal rather than the normal model the farther the *CV* gets above 10%.
3. Youden two-sample chart. Figure 3 shows the type of patterns to expect from lognormal data for two different degrees of between-laboratory variation. One should expect fan-shaped patterns with points concentrated in the First quadrant, rather than the more balanced elliptical patterns characteristic of the normal distribution [5].
4. Outlier tests. The commonly used outlier tests are based on the normal distribution, which is symmetric, so applying such tests to lognormal data tends to give erroneous results. For example, when Grubbs test is applied to untransformed lognormal data, there is a tendency to miss real lower-tail outliers and to find too many upper-tail “outliers.” The problem grows as the *CV* increases, but it is easily cured by applying the test to log-transformed data.

In conclusion, the lognormal distribution appears to be the appropriate model for some methods of trace analysis. As the *CV* increases, it becomes more important to use this model when it is appropriate; its use does not require unmanageable changes in analytical QC practices.
